# Practical Medicine and Pathology

**Published:** 1875-11

**Authors:** 


### V. Practical Medicine and Pathology.

1. *Non-Malarious Origin of Intermittent Fevers.* Thos. H. Streets, Asst. Surgeon, U. S. N. (*Phil. Med. Times*, Sept. 18th.)

Several cases of intermittent fever occurred on board the steamer "Portsmouth" during her recent cruise, under such conditions as led to the belief that they were caused by other agencies than malaria. He thinks these were atmospheric changes, thermometric and barometric, as these always preceded or accompanied the attacks. Passing from the tropics to latitude 40° S., and encountering fogs and damp, chilly weather, cases of intermittent fever appeared. When cruising eastward of the Sandwich Islands, the steamer passed from mild weather into a cold polar current, with moisture from condensation, cases of intermittent suddenly appeared on board. At the same time there appeared cases of rheumatism, bronchitis, tonsilitis and neuralgia. At this time the ship was hundreds of miles beyond the limit of the malarial influence of the shore.

To the windward of Honolulu are numerous fields of *taro* root, which from being composed of soft mud are prolific of malaria, yet intermittent fever is rare in the town. There were no cases on board the ship while she lay at H., except when the trade winds were interrupted and the south wind brought rain. At such times there occurs influenza and tonsilitis, and the natives call these south winds, "Kona," meaning "sick-winds."

2. *Complete Gangrene of the Left Kidney.* AVEZOU. (*Phila. Med. Times; Le Progrès Médical.*)

A man of 79, with left-sided pneumonia, died in eight days. He had had for years lumbar pain on the left side, but never other symptoms that indicated kidney disease. An autopsy revealed hepatization of the left, and congestion of the right lung, with atheroma of the aorta. The right kidney appeared normal, the left was black, friable and fetid ; the tissues surrounding this kidney were dark colored, its ureter was patulous, and its artery was nowhere obliterated. The microscope revealed interstitial nephritis and the alterations due to it in both kidneys.

3. *The Modus Operandi of Yellow Fever Poison.* G. M. STERNBERG, M.D. (*New Orleans Med. and Surg. Jour.*, July, 1875.)

The theory of zymosis is objected to, as wanting in substantial proof.

The hypothesis is advanced "that the first and essential effect of the yellow fever poison is to produce a disturbance of the functions of the sympathetic nervous system, and that the grave changes in the blood which occur in the course of the disease are secondary in their nature, and result from the arrest of vital processes (nutrition, excretion, secretion) presided over by the sympathetic." While during the disease blood changes occur that may account for some of the symptoms, Dr. S. is not aware that prior to, or at the time of the invasion, any such changes have been discovered as would account for the pronounced and sudden symptoms. He believes the liability to an attack as well as its severity, is largely due to the dose of the poison taken, and not wholly to the age, sex, habits, etc.; and this position, if true, is against the zymotic theory. For proof that the poison is capable of self-multiplication outside the body, he quotes Ziemssen and Reynolds, and that it is a vegetable fungus, he thinks the supposition very strong. Various poisonous fungi are known to produce their symptoms at a variable time after being taken. The poisonous mushroom sometimes does not lead to severe symptoms for thirty hours after being eaten. The variable time after known exposure to yellow fever poison, at which the attack occurs, has always been a notable feature in this affection. The author thinks it most probable that the fungi act on the ganglionic centres indirectly, by first impressing the peripheral distribution of the sympathetic in the alimentary mucous membrane. Too small a quantity of the fungi for a poisonous effect, might easily find on the mucous membrane a nidus for multiplication until sufficient to do injury had been produced. Extensive quotations are made from standard authors on physiology and on yellow fever, to show that many of the symptoms of this disease are such as are produced by known disturbances of the sympathetic system, by various experiments, drugs, etc. Among the symptoms touched upon are, chill, rise of temperature, headache, rapid breathing and pulse, redness of the mouth and throat, epigastric tenderness, vomiting, diminished urine. These symptoms occur early in the disease; many of those occurring later may easily be produced by the disturbances in the functions taking place at beginning.

Finally, the evidence is thought convincing that the sympathetic is involved in yellow fever; that there is no proof of blood changes occurring prior to such involvement of the sympathetic, which later marks the outbreak of the disease; that the effect of the poison seems to be "a paralyzing one," causing phenomena similar to those following a division of the sympathetic in any part of the body; and that the question whether the blood is, with the sympathetic, also primarily affected, is still *sub judice.*

4. *Some Practical Observations on Exophthalmic Goitre and its Treatment.* Robert Bartholow, M.D. (*Chicago Journal of Nervous and Mental Diseases*, July, 1875.)

This is a short paper, read before the American Medical Association. Attention is called to the fact that of the three distinctive characteristics of this affection, increased action of the heart, thyroid enlargement and exophthalmos, one may occur—the first preferably—without the others. Dr. B. thinks many of the cases of rapid heart's action with paroxysmal palpitation are of this sort, and similar in pathology to this disease. He thinks also, many cases of so-called *goitre* "are really examples of Graves' disease—the enlarged thyroid only attracting attention." The distinction between the two is, that in goitre there is hypertrophy or hyperplasia, or both, of gland elements, while in Graves' disease the thyroid enlargement is largely due to dilatation of vessels and to hyperkinesis of the heart. In the latter, the enlargement is fluctuating; in the former, it is more continuous.

When the exophthalmos is only slight, the method of examination of von Graefe is commended. This consists in having the patient simply look down, when, if the condition exists at all, "a white rim" of the sclerotica appears below the upper lid and abôve the iris.

Three illustrative cases are reported. One was that of a woman of 32 years; there were paroxysms of palpitation; constant elevation of pulse-rate; intercostal neuralgia, often very troublesome; but only slight exophthalmos, and less enlargement of the thyroid.

Chalybeates and other tonics wholly failing, the constant galvanic current, with 20 cells, was resorted to regularly, the negative pole at the epigastrium and the positive against the neck. Perfect recovery resulted.

Another case was that of a woman of 54 years. There was goitre of many years standing, palpitation and increased action of the heart, large pigmentary discolorations of the skin, in spots about the neck, the sternal region and (symmetrically) on the dorsal aspect of the hands, and cachexia, but no appearance of exophthalmos.

The treatment was the same as in the first case, and recovery was nearly complete in three or four months.

The other case was that of a woman fifty years old. Here a considerable exophthalmos had appeared suddenly after a night of great anxiety. Rapid heart's action had occurred occasionally before, and palpitation frequently followed. There was an hardly perceptible increase in the thyroid region. She was anæmic, menstruated regularly, and at these periods the protrusion of the eyes was increased.

She was treated with the constant current in the manner described. Gradual improvement followed, and recovery was complete within a year.

Dr. B. thinks *exophthalmic goitre* is, according to present knowledge, one of the disorders of the sympathetic.

5. *Hæmaturia.* DAVID CHOATE, M.D. (*Boston Med. and Surg. Journal*, Sept. 16, 1875.

An interesting case is here reported of the disorder named, in a stout laborer, 40 years old. The discharge of blood was almost continuous for about nine months, it appearing in every specimen voided. The quantity lost could not be given, but the urine was invariably colored deep red, and the microscope showed abundant red corpuscles; the blood was always mixed with the urine, never coming clear or in coagula. No mucus, pus or casts, could be detected; and only so much albumen as was thought due to the elements of the blood.

Increasing debility compelled the patient, near the close of the sickness, to take to his bed. The only remedy that appeared to diminish the flow was gallic acid in five grain doses, every two hours.

Finally, hypodermic injections of ergotine were resorted to, one-fifth grain twice daily. In three days the dose was increased to one-fourth grain. In five days vomiting came on, lasting for several hours, and the ergotine was withheld. Suddenly the urine became clear, and remained so. The next day the patient had a sharp attack of pain in the course of the right ureter, "followed by the passage of a small coagulum, apparently moulded by the ureter." Recovery closely followed.

The pathology of the case was obscure. It was learned that the patient had for some time before the hæmaturia, been failing in strength and appetite, and had resorted to regular rations of alcoholic stimulants.

6. *The Influence of the Lower Organisms in the production of Infectious and Contagious Diseases.* (*Med. Times*, Phila.)

In a series of three articles in the *Times*, the above named subject is reviewed, After an extended reference to the studies and investigations that have been made on the subject, the reviewer quotes from Birch-Hirschfield, who, in a review of our present knowledge of the bacterial origin of disease, speaks as follows: "In a majority of diseases, the fungous origin of which has been made the subject of inquiry, we possess little or no exact proof of a parasitic cause, not even in the case of malignant pustule, or that of relapsing fever. But the *probability* that bacteria are not insignificant associates of these pathological processes, is, to an unprejudiced observer of the facts recently discovered, very strong, and the same may be said of the diseases previously mentioned." "Much which we already know regarding various circumstances connected with the life and development of bacteria, agrees singularly well with the ideas impressed upon our minds when we endeavor to form a conception of the infectious material of epidemic disease."

"It must be conceded, however, that so far as most of the forms of bacteria are concerned, investigation shows that, if they are not received in too overwhelming quantity, the *healthier* organism offers them an almost absolute resistance." "Proof also is available in the case of many diseases for the assertion that *those only are attacked by the disease who were sick already.*"

